# Mie light scattering calculations for an Indian age-related nuclear cataract with a high density of multilamellar bodies

**Published:** 2008-03-24

**Authors:** Kurt O. Gilliland, Sonke Johnsen, Sangeetha Metlapally, M. Joseph Costello, Balasubramanya Ramamurthy, Pravin V. Krishna, Dorairajan Balasubramanian

**Affiliations:** 1 Department of Cell and Developmental Biology, University of North Carolina, Chapel Hill, NC; 2 Department of Biology, Duke University, Durham, NC; 3 L.V. Prasad Eye Institute, Hyderabad, India

## Abstract

**Purpose:**

Multilamellar bodies (MLBs) are lipid-coated spheres (1–4 µm in diameter) found with greater frequency in the nuclear region of human age-related cataracts compared with human transparent lenses. Mie light scattering calculations have demonstrated that MLBs are potential sources of forward light scattering in human age-related nuclear cataracts due to their shape, size, frequency, and cytoplasmic contents, which often differ in refractive index from their surroundings. Previous studies have used data from several non-serial tissue sections viewed by light microscopy to extrapolate a volume and have assumed that MLBs are random in distribution. Currently, confocal microscopy is being used to examine actual tissue volumes from age-related nuclear cataracts and transparent lenses collected in India to confirm MLB shape, size, frequency, and randomness. These data allow Mie scattering calculations to be done with directly observed MLBs in intact tissue.

**Methods:**

Whole Indian donor lenses and Indian lens nuclei after extracapsular cataract extraction were immersion-fixed in 10% formalin for 24 h and in 4% paraformaldehyde for 24 h before sectioning with a Vibratome. The 160 µm thick sections were stained for 24 h in the lipid dye DiI (1,1’-dilinoleyl-3,3,3′,3′ tetramethylindocarbocyanine, 4-chlorobenzenesulfonate), washed, stabilized in Permount under coverslips and examined with a Zeiss LSM 510 confocal microscope. Individual volumes of tissue (each typically 500,000 µm^3^) were examined using a plan-apochromat 63X oil (NA=1.4) lens. Other lenses were prepared for electron microscopy and histological examination using previously described procedures.

**Results:**

Analysis of tissue volumes within Indian age-related nuclear cataracts and transparent lenses has confirmed that most MLBs are 1–4 µm in diameter and typically spherical with some occurring as doublets or in clusters. Most Indian cataracts and transparent lenses are similar to samples obtained in the United States. One cataract contained as many as 400,000 MLBs per mm^3^ –100 times more than in cataracts collected in the United States. Pairwise distribution analysis has revealed that MLBs even in this exceptional case are found with a distribution that appears to be random. Mie calculations indicate that more than 90% of the incident light could be scattered by the high density of MLBs.

**Conclusions:**

An important finding was that one advanced Indian cataract contained many more MLBs than cataracts examined from India and previously from the United States. This indicates that specific conditions or susceptibilities may exist that promote the formation of excessive MLBs. Based on the extremely high frequency, as well as their spherical shape, large size, and apparent random distribution, the MLBs are predicted according to Mie light scattering calculations to cause high amounts of forward scattering sufficient to produce nuclear opacity.

## Introduction

The most common cause of blindness is cataract [1,2]. In India, blindness due to cataract is significantly greater than in western populations according to recent studies [3-8] with nuclear opacities being most common [3]. For example, a recent study in India has demonstrated that the prevalence of blindness is over 6%, and of those who are blind, bilateral cataract is the cause for almost 80% of that blindness [9].

Therefore, numerous initiatives to provide successful and sustainable cataract services and to prevent future problems have been developed by organizations such as the International Agency for Prevention of Blindness and the World Health Organization (WHO). These global efforts, including the National Programme for Control of Blindness [3,8] and “Vision 2020: Right to Sight” [10], have been responsible for cataract surgeries in India being performed at a rate of 4.5 million per year. By 2020, it is projected that blindness due to cataract will no longer be a major concern in India [1,7,11,12]. Such improvements in medical care and nutrition will likely reduce the number of those with cataract in the population initially, but at the same time, the population will age. With this growth in the elderly population, the number of those susceptible to the development of age-related nuclear cataract will increase [3,13].

Despite general improvements to access medical care in India, there is still inadequate delivery of cataract surgical services to the rural population and to disadvantaged groups [3,9,14]. A recent study showed that only 12% of those blind in a particular region of south India received surgery [9]. Currently, the prevalence of cataract in India is unexplained, although it is thought that contributing factors may be exposure to ultraviolet radiation while working outdoors without visual protection [15] or poor nutrition [16,17]. Therefore, it is necessary to identify both the risk factors for cataract and the cellular and molecular pathology of cataract so that the disease may be prevented, delayed, or one day even cured without surgery.

Cataract is a multifactorial disease, and lenses with cataracts may have many ultrastructural sources of light scattering. For age-related nuclear cataracts, high-angle scattering where light scatters backward toward the clinician observing with a slit lamp results in less light reaching the retina and therefore a dimmer image [18]. High-angle scattering may in part be caused by small (<0.1 µm in diameter) high-molecular-weight aggregates, which are proposed to form from cytoplasmic proteins that have undergone oxidative damage [19-24]. However, during the early stages of nuclear cataract formation, the fiber cell cytoplasm is smooth and homogeneous by light and electron microscopy [25,26] with only slight texturing present in a few cases [27]. Thus, the high-molecular-weight aggregates do not create significant fluctuations in refractive index, and some other source of scattering must exist [28]. Low-angle scattering, on the other hand, where light scatters in the forward direction, is likely to affect image formation at the macula [29]. Although it is quite difficult to measure in vivo, large fluctuations in density or large particles would produce forward scattering. Based on mathematical modeling [30,31], microscopic observations [32,33], and Mie scattering predictions [33,34], it has been suggested that a significant source of forward light scattering is likely to be large, rare, spherical particles within the cytoplasm. Because these structures are coated with multiple layers of thinly-spaced lipid membranes, they are known as multilamellar bodies (MLBs) [32-34].

MLBs are spherical in shape with an average diameter of 2–3 µm and are composed of a cytoplasmic core surrounded by anywhere from 3 to 10 membrane layers. The cytoplasm of the interior of the MLB often stains differently from the surrounding cytoplasm, and the membranes forming the coat of the MLB are thinly-spaced with an intermembrane spacing of less than 5 nm [32-34]. Most importantly, MLBs are found with 7.5 times greater frequency in the immature cataracts observed in the United States than in transparent lenses [32,33].

In India, age-related prevalence of cataract is significantly greater than the prevalence in the United States [3,35,36] and affects individuals younger than those in the United States [4,5,37]. Furthermore, the cataracts are more advanced than in those in the United States. Therefore, it was hypothesized that some Indian cataracts might contain significantly more MLBs than those previously analyzed in the US.

Mie’s solution to Maxwell’s electromagnetic equations [38-41] is used to predict the contribution of the MLBs to the forward scattering of light. Ideally, the particles should be spherical in shape, separated by distances greater than a few wavelengths of incident light, and random in distribution. Since MLBs meet these requirements, the Mie scattering theory is appropriate for describing forward light scattering caused by MLBs. The purpose of this study was to compare aged transparent lenses and advanced cataractous lenses collected in India to determine causes of light scattering. The initial examination revealed a similar frequency and appearance of MLBs in these specimens compared to immature cataracts previously analyzed in the United States. The predicted scattering from the immature United States cataracts was less than 20% of the incident light [33,34]. In one exceptional case, a high density of MLBs was observed and may characterize some advanced cataracts from India. For such cataractous nuclei containing a large density of MLBs, nearly all the incident light was predicted to be scattered by the MLBs.

## Methods

### Lenses

During a two-week period, a total of 44 lens samples were collected and processed at the L.V. Prasad Eye Institute (LVPEI, Hyderabad, India) following the tenets of the Declaration of Helsinki and using procedures approved by the Institutional Review Board of the University of North Carolina at Chapel Hill (UNC). Only limited information was provided without patient identifiers including age, gender, cataract grade, and whether the patient was diabetic. Sixteen lens samples were excluded because the processing was not ideal, they were from diabetic patients, or they were not a suitable cataract type. For this study, seven transparent donor lenses were obtained from the Ramayamma Eye Bank within LVPEI and 22 cataractous nuclei were obtained from the operating suites following extracapsular extraction. None of the intact donor lenses (average equatorial diameter of 9.4 mm) showed signs of opacification by handheld slit lamp examination or distortion of a mesh viewed through the lens in transmitted light. All of the cataractous nuclei were from mature cataracts that did not pass enough light to form an image and were dark yellow to densely brunescent in color. These nuclei (average equatorial diameter of 7.7 mm) probably represented advanced age-related nuclear cataracts from patients that were blind before surgery. All lens samples were processed by the three investigators from UNC, using the same model tissue slicer, chemicals shipped from United States sources, and protocols refined at UNC in earlier studies [32,33]. Many lenses were processed simultaneously, and the procedures required several days to complete, resulting in fixed, inert embedded material, which was shipped with permission of the Indian Ministry of Health back to UNC for microscopic analysis. This elaborate process ensured that the highest level of ultrastructural preservation was achieved and that samples could be directly compared to those of previous studies using immature nuclear cataracts. Selected lens samples were processed separately for dye staining used in scanning laser confocal microscopy. These procedures produced a unique set of lens samples prepared from fresh fully opaque advanced nuclear cataracts.

### Confocal microscopy

**Figure 1:**
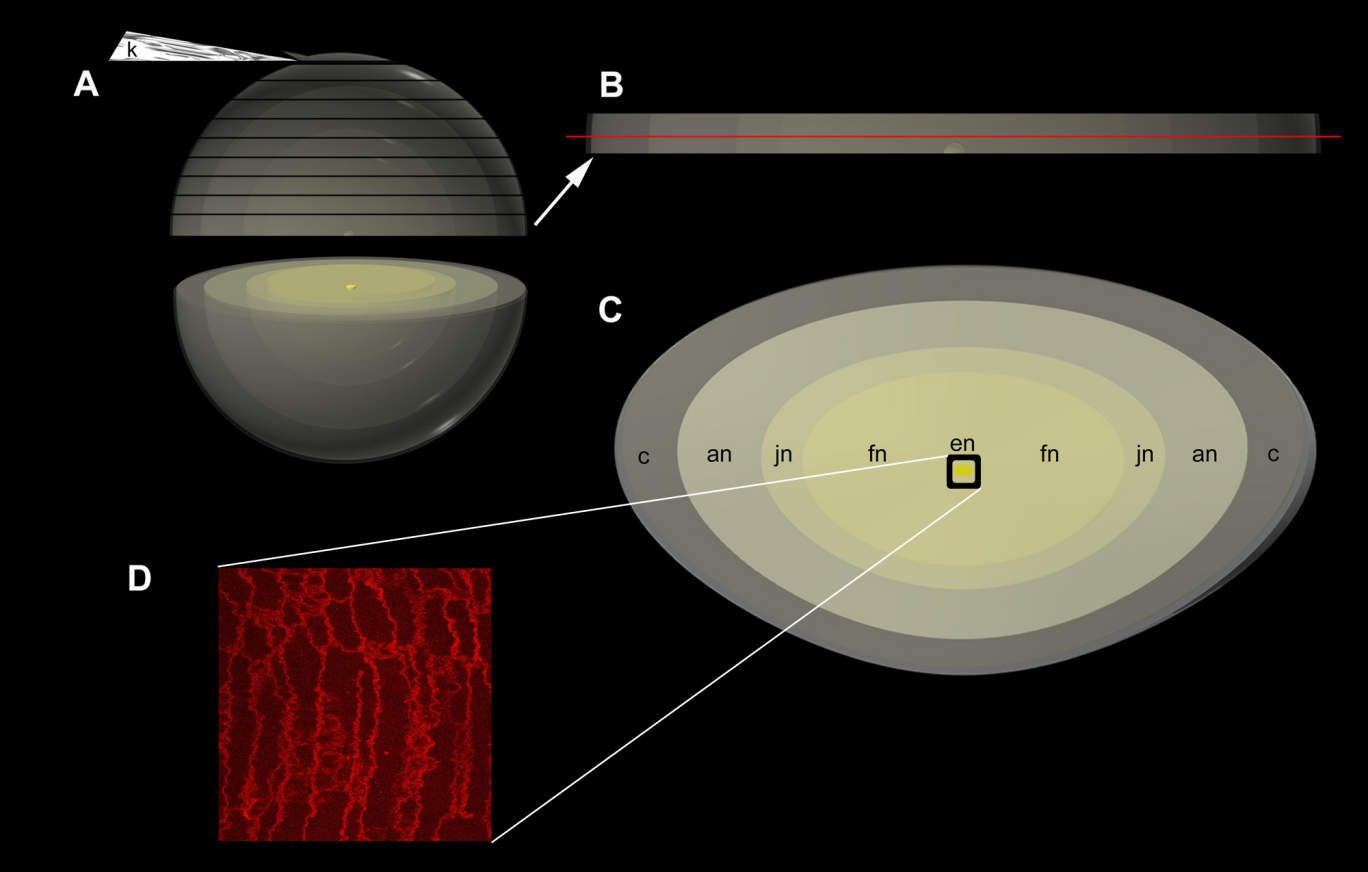
Methods. Transparent lenses and age-related cataractous nuclei were immersion-fixed and sectioned with a Vibratome (**A**). The 160 µm thick sections were stained with the lipophilic dye, Fast DiI, washed with ethanol, and transferred to glass slides with Permount and coverslips. For analysis, sections at or near the center of the lens were used so that all developmental regions could be identified (**C)**, including the embryonic nucleus (en), fetal nucleus (fn), juvenile nucleus (jn), adult nucleus (an), and cortex (c). Samples were examined with laser scanning confocal microscopy (**D**). Individual volumes of tissue from each Vibratome section were visualized. The red line in (**B**) indicates an example of one focal plane that was used.

Two non-diabetic, non-cataractous, aged, transparent donor human lenses (both at the age of 45) and five cataractous human nuclei (ages 60, 60, 66, 70, and 76) from extracapsular cataract extractions were obtained from the surgical unit and eye bank, respectively, at the LVPEI. The anterior-to-posterior thickness and equatorial diameter of each lens or nucleus were measured so that the center could be determined. Lenses and nuclei to be examined with confocal microscopy were immersion-fixed in 10% formalin for 24 h and in 4% paraformaldehyde for approximately 24 h. Specimens were then mounted with cyanoacrylate glue onto a metal sectioning tray, covered with warm 2.5% agar, submerged in 1X phosphate buffered saline (PBS) at 10 °C, and sectioned using a vibrating knife microtome (Model 1000; Vibratome, St. Louis, MO) at a speed of approximately 0.2 mm/s, an amplitude of 6, and a cutting angle of 12° (Figure 1A).

Vibratome sections (each 160 µm thick) at and near the center of each lens were collected, washed for 5 min in a 1:1 solution of 1X PBS and 50% ethanol, and then stained for approximately 18 h in the lipid dye, Fast DiI (1,1’-dilinoleyl-3,3,3′,3′ tetramethylindocarbocyanine, 4-chlorobenzenesulfonate; Molecular Probes Inc., Eugene, OR), at a concentration of 0.2 mM following a procedure modified from previously published protocols [42]. The combination of prefixation and lengthy staining allowed the dye to penetrate completely to the core of each 160 µm thick section. Sections were then washed for 5 min in 100% ethanol and stabilized in Permount under #1 coverslips, each enhanced with two 0.17 mm thick glass platforms between which the 160 µm thick sections were situated. Optical sectioning of DiI-stained samples on a Zeiss LSM-510 (Carl Zeiss Inc., Thornwood, NY) laser scanning confocal microscope with a plan-apochromat 63X oil (NA=1.40) lens was performed to examine the tissues. The red line in Figure 1B indicates an example of one focal plane that was used in a given section, which was from a location at or near the center of the lens, so that all developmental regions could be identified (Figure 1C). Individual volumes of tissue (from multiple z-series totaling approximately 500,000 µm^3^) from a Vibratome section were able to be visualized (Figure 1D). Volumes were reconstructed in three-dimensional (3D) views using Imaris 3.0 (Bitplane Scientific Solutions, Zurich, Switzerland). Because the MLBs were brighter than adjacent membranes, they could be located automatically by thresholding. Marker particles were inserted to give 3D views of the distribution [33] and exact coordinates of each particle.

### Bright-field light microscopy

Vibratome sections approximately 200 µm thick were processed as previously described [33]. Briefly, thick sections were fixed for 12–18 h in 0.5% glutaraldehyde, 2% paraformaldehyde, and 1% tannic acid in 0.1 M cacodylate buffer (pH 7.2). Sections were then washed with distilled water for three 15 min washings, stained in 2% aqueous uranyl acetate in the dark for 60 min, washed with distilled water once for 10 min, and dehydrated through a graded ethanol series. Sections were infiltrated and embedded in LR White resin (Electron Microscopy Sciences, Ft. Washington, PA) from which histological sections (0.7 µm thick) of the nuclei were cut along the optic axis and mounted on glass slides. Mounted sections were stained with toluidine blue oxide (TBO), coverslipped, and examined with a Leica DMR bright-field microscope (Leica, Solms, Germany). Digital images were collected with a Leica DC500 12 megapixel digital camera (Leica).

### Transmission electron microscopy

As described previously [32], Vibratome sections approximately 200 µm thick were fixed for 12–18 h in 2.5% glutaraldehyde, 2% paraformaldehyde, and 1% tannic acid in 0.1 M cacodylate buffer (pH 7.2). Sections were then washed with 0.1 M cacodylate for three 15 min washings, treated with cold 0.5% osmium tetroxide for 60 min, washed with distilled water for three 15 min washings, washed once with 50% ethanol for 5 min, stained in 2% uranyl acetate (ethanol-based) in the dark for 30 min, and dehydrated through a graded ethanol series. Sections were infiltrated and embedded in an epoxy resin. Thin sections (50–70 nm) were cut with a diamond knife (Diatome USA, Fort Washington, PA) from mesas raised along the embryonic nucleus of the optic axis and stained with uranyl acetate and lead citrate for viewing at 80 kV on a FEI-Philips Tecnai 12 (FEI Company, Hillsboro, OR) transmission electron microscope.

### Quantitative analysis

For each lens, several small volumes, typically totaling approximately 500,000 µm^3^, were thoroughly searched by confocal microscopy and MLBs were counted. MLB frequency was expressed as a ratio of MLBs per cubic millimeter. To test the randomness of MLB distribution, each MLB’s position within a specific rectangular volume was determined. The volume chosen for the calculation was derived from a z-series of 80 sections that were 0.5 µm thick with lateral dimensions of 73 µm by 73 µm, giving a total volume of approximately 210,000 µm^3^. A cumulative histogram of the distances from each MLB to its nearest neighbor was then generated. This histogram was compared with the upper and lower envelopes of histograms from 100 and 1000 simulations of nearest neighbor distances of the same number of particles of the same size positioned randomly within the same volume using Monte Carlo methods [43] incorporating routines within MATLAB (version 6.2, MathWorks, Natick, MA). This approach overcomes the uncertainty of detecting nearest neighbors of MLBs close to the edge of the sampling volume, because potentially close MLBs just outside the sampling volume would not be detectable using analytical methods [43]. For a set of Monte Carlo simulations, the upper and lower limits at each fraction of MLBs in cumulative histograms defines the envelopes, which show less fluctuations as the number of simulations is increased. A random distribution of real particles will fall within the envelopes.

Light scattering can be calculated using the Mie scattering theory for coated spheres as has been done in previous publications [33,34]. Briefly, the algorithms of Mätzler [44] were employed using MATLAB (version 6.2, MathWorks, Natick, MA) with key input parameters of MLB average diameter, wavelength of incident light, refractive index of the MLB interior, and refractive index of the cytoplasm surrounding the MLBs.

## Results

Previous studies have determined the distribution of MLBs in histological sections of immature cataracts from the United States at the equatorial plane [32] and along the optic axis [33] and used the Floderus equation [44] to predict how many of the spherical structures might be present in a true volume of tissue. The current study utilizes confocal microscopy to examine a true volume of tissue employing serial optical sections of transparent lenses and advanced cataracts from India.

**Figure 2:**
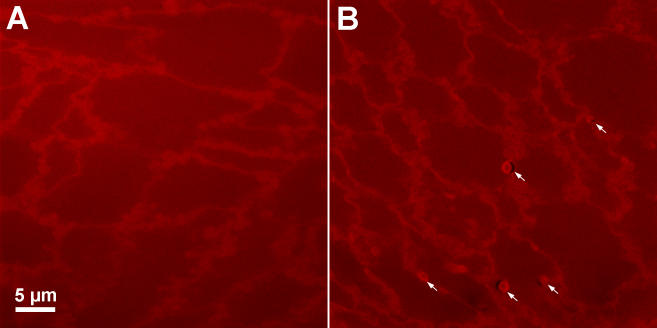
Confocal images of transparent and cataractous Indian lenses. While transparent Indian lenses typically show no MLBs in individual optical sections (**A**), the exceptional Indian cataractous nucleus shows several MLBs (arrows) within a similar field of view in nearly every optical section (**B**).

While the transparent Indian lenses do contain rare MLBs, most optical sections display no MLBs (Figure 2A). In a volume of approximately 210,000 µm^3^ (80 sections, each 0.5 µm thick with lateral dimensions of 73 µm by 73 µm), only one to three MLBs can usually be observed. The typical Indian age-related cataract shows MLBs that are similar in density to those of United States cataracts [32,33]. An exceptional Indian advanced cataract displayed numerous MLBs within each optical section (Figure 2B).

**Figure 3:**
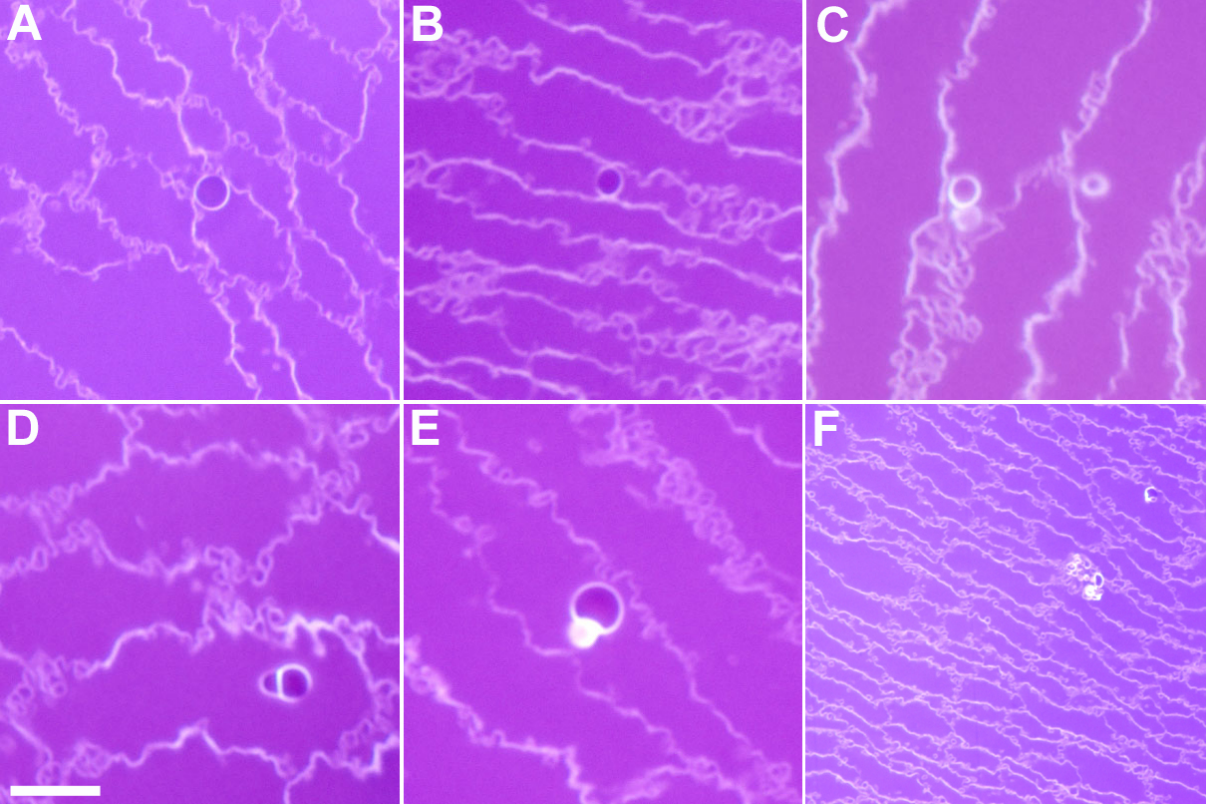
Shape and size of MLBs. As seen in bright-field microscopy (**A**-**F**), multilamellar bodies (MLBs) from age-related nuclear cataracts are roughly spherical in shape and approximately 2.1 µm in diameter. (**D**) and (**E**) demonstrate that MLBs sometimes occur in a doublet formation, and (**F**) depicts a rare situation where a cluster of small particles are gathered together. Scale bar=5 µm.

It has been suggested in initial studies of MLBs that they are spherical and on average 2.4 µm in diameter [32,33]. To confirm MLB morphology and to measure the diameter accurately, laser scanning confocal microscopy (Figure 2B) and bright-field microscopy (Figure 3A-F) were used to examine numerous MLBs from age-related Indian nuclear cataracts. Optical sections confirm that MLBs are roughly spherical in shape when they occur in isolation (Figure 2B and Figure 3A,B), although they sometimes occur in a doublet formation (Figures 3C-E) or as a cluster of small particles (Figure 3F). The diameters of the MLBs in the Indian cataract with a high MLB density was 2.1 µm (n=43).

**Figure 4:**
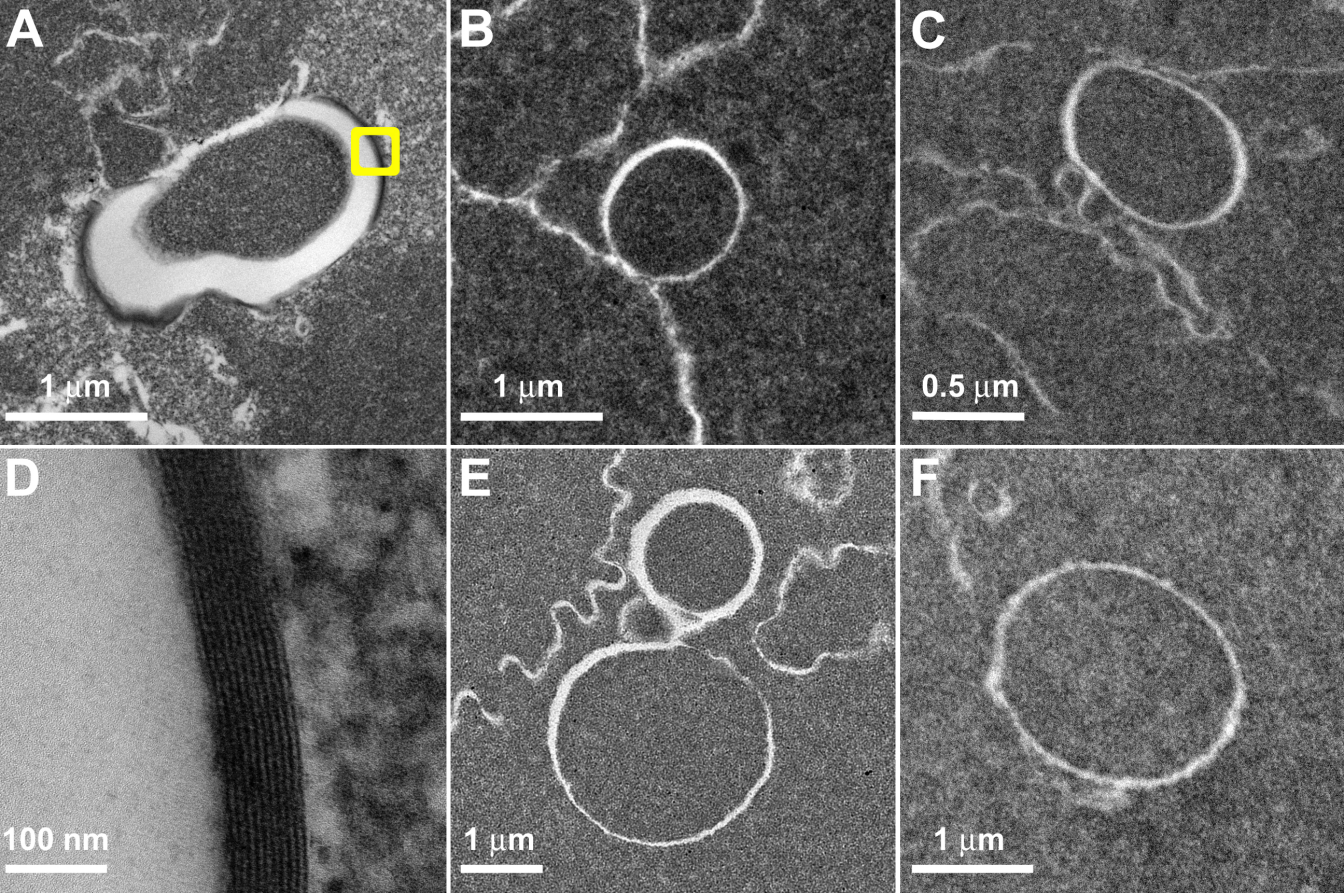
Membranes of MLBs. As shown in the transmission electron micrographs, MLBs from age-related nuclear cataracts often appear circular (**B**, **C**, and **F**) with an occasional doublet formation (**E**). MLBs are covered with multiple layers of lipid membranes, although these membranes can barely be visualized at low magnification (**A**) in this donor lens. With proper tilt (25°-40°) and high magnification (**D**, from the inset box in **A**), the 10 membranes appear multilamellar with thin spacing (4.5 nm).

Transmission electron microscopy (TEM) was used to examine the membranes of MLBs. An MLB may contain anywhere form 3–10 lipid layers within the space forming its coat, although these membranes are not typically visible (Figure 4B,C,E,F). Figure 4A depicts a donor lens in which the membranes can barely be seen at low magnification. With proper tilt and high magnification (Figure 4D), the 10 membranes forming the multilamellar coat can be visualized and are seen to have uniformly thin spacing (approximately 4.5 nm).

TEM thin sections and bright-field light microscopic histological sections were examined qualitatively for the distribution of MLBs. Thin sections were searched at low magnification of 1000X-4000X for all 17 lenses prepared for TEM. TBO-stained histological sections from seven of these lenses were examined at 630X and 1000X by bright-field microscopy. The objective was to locate MLBs, which were visible in every cataractous lens examined, and to estimate the density of MLBs based on the number within each section mesa and the distance between adjacent MLBs. The appearance of the MLBs (Figure 3 and Figure 4) and their approximate distribution in the advanced Indian cataracts were similar to those reported previously for United States immature cataracts [32-34]. Although the density measurements were not as extensive as in previous studies [32,33], it was clear that none of the lenses contained numerous MLBs that would produce multiple MLBs in a low magnification view as in Figure 2B.

In previous studies, it was assumed that MLBs have a random distribution [32-34]. This assumption was reasonable, as MLBs appeared to be uniformly scattered throughout the embryonic and fetal nuclear regions and it was very rare to observe two MLBs within the same field of view where magnifications of 400X or higher were needed to clearly identify the particles as MLBs. The average separation was well in excess of 10 µm, which is very much greater than the wavelength of visible light (0.4–0.7 µm). To calculate the total scattering from all the MLBs, one must assume that they are sparsely and randomly distributed and therefore that they scatter independently. In this study, it was possible to test this assumption, because the distribution of MLBs in real tissue volumes could be determined.

**Figure 5:**
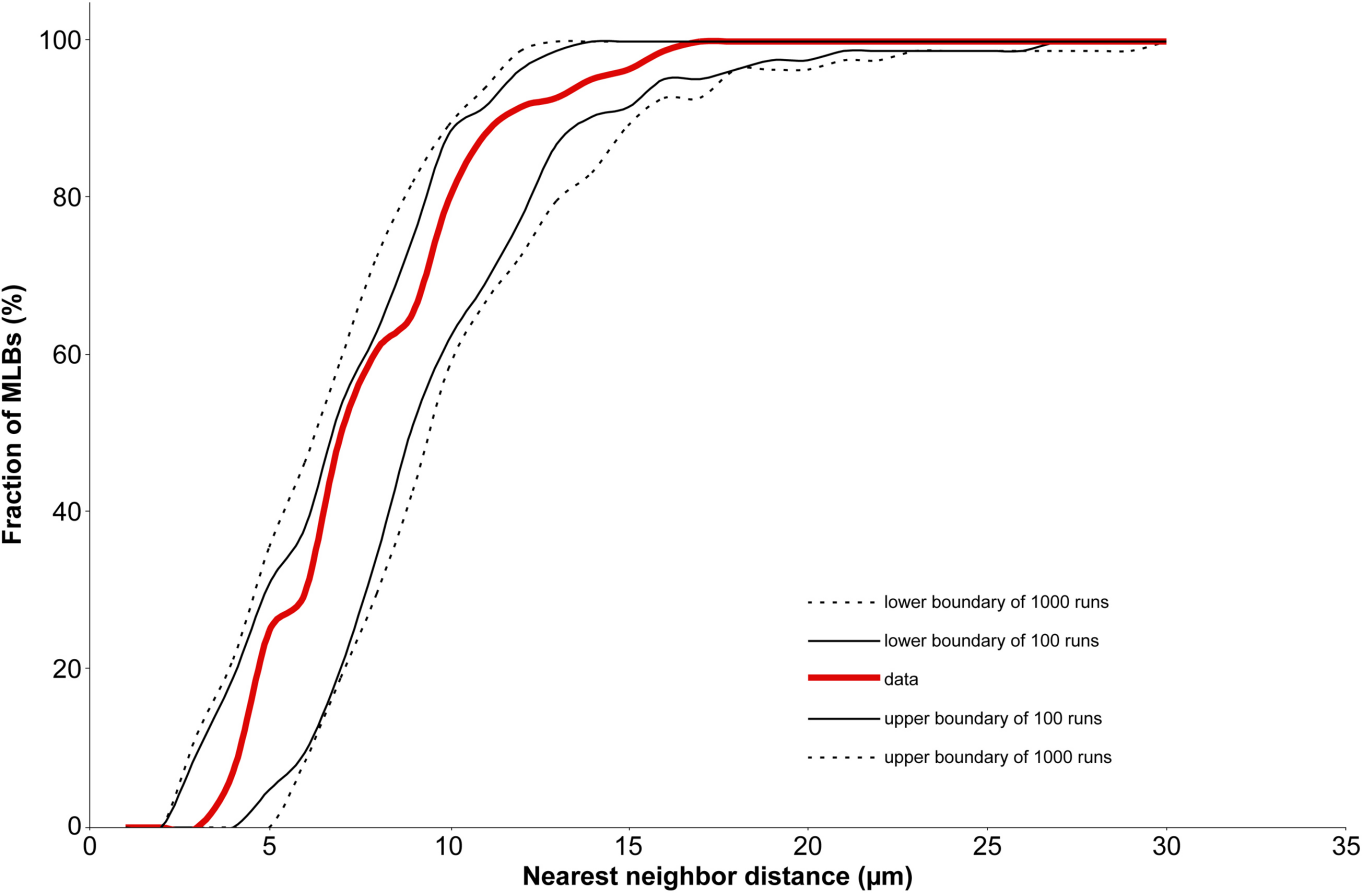
Randomness of MLBs by Monte Carlo analysis. Each position of 92 MLBs within a rectangular volume was determined with x, y, and z coordinates. The distance between each MLB and all other MLBs was then measured, and the nearest neighbor distances are displayed on this cumulative histogram (red line) and compared with the upper and lower bounds of the histograms created by 100 and 1000 random repetitions (solid and broken lines) of analyzing the same number of random particles in the same size volume. This pairwise distribution analysis shows that MLBs are found with a random distribution that is within the ranges determined by the Monte Carlo trials.

The evaluation of randomness of the distribution of MLBs is illustrated for the exceptional Indian nuclear cataract with a high density of MLBs using a confocal z-series reconstructed volume. Coordinates in *x*, *y*, and *z* were obtained for each of the 92 MLBs within a rectangular volume of about 210,000 µm^3^, allowing the distance of each MLB to its nearest neighbor to be measured. Then, in a computer simulation, 92 particles of the same diameter were randomly positioned within an identical rectangular volume and nearest neighbor measurements were made; this simulation was repeated 100 times and then 1000 times using Monte Carlo methods. For each simulation, the upper and lower limits of the nearest neighbor distances were used to produce envelopes that described a range of distances corresponding to random distributions. The cumulative histogram of the MLBs falls within the envelopes, providing strong evidence that the distribution of MLBs analyzed is random (Figure 5).

**Figure 6:**
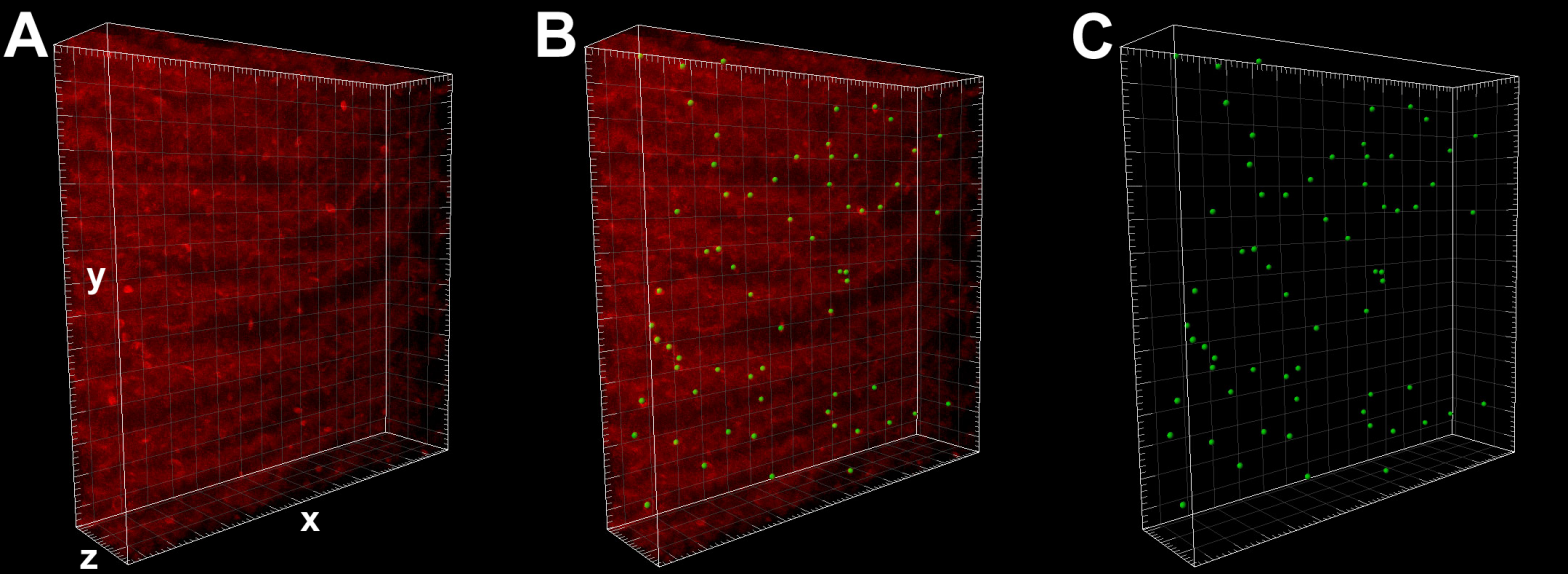
Distribution of MLBs. **A**: An example of one of the many volumes examined is shown. This particular volume from an age-related nuclear cataract is 73 µm (x) by 73 µm (y) by 16 µm (z) for a total volume of approximately 85,000 µm^3^. The z distance of 16 µm resulted from the stacking of 40 optical sections of 0.4 µm each. MLBs appear as bright spots as their lipid coatings are labeled well by the lipophilic DiI. **B**: To facilitate the viewing of MLBs, a sphere has been placed manually over each MLB with the aid of thresholding to locate most of the MLBs. **C**: With the membranes removed, it can be seen that 64 MLBs are located within this volume of 85,000 µm^3^ – a frequency of 753,000 MLBs per mm^3^.

While many Indian age-related cataracts contained MLBs with the same frequency as those previously analyzed in the United States [32,33], the one Indian lens examined with an exceptionally high number of MLBs is emphasized here. The MLBs within a volume similar to that described above to establish the random distribution mathematically can be used to directly visualize the MLB distribution. The MLBs appear as bright spots because their lipid coatings, which are labeled by the lipophilic dye, DiI, are displayed in a reconstructed volume of approximately 85,000 µm^3^ (Figure 6A). To facilitate the viewing and counting of MLBs, a sphere has been placed over each MLB (Figure 6B). After the membranes are removed by thresholding, it can be seen that 64 MLBs are located within this volume of 85,000 µm^3^ – a frequency of 753,000 MLBs per mm^3^. This frequency, while exceptional even among Indian cataracts, is much greater than that of the most advanced cataract analyzed in the United States.

**Figure 7:**
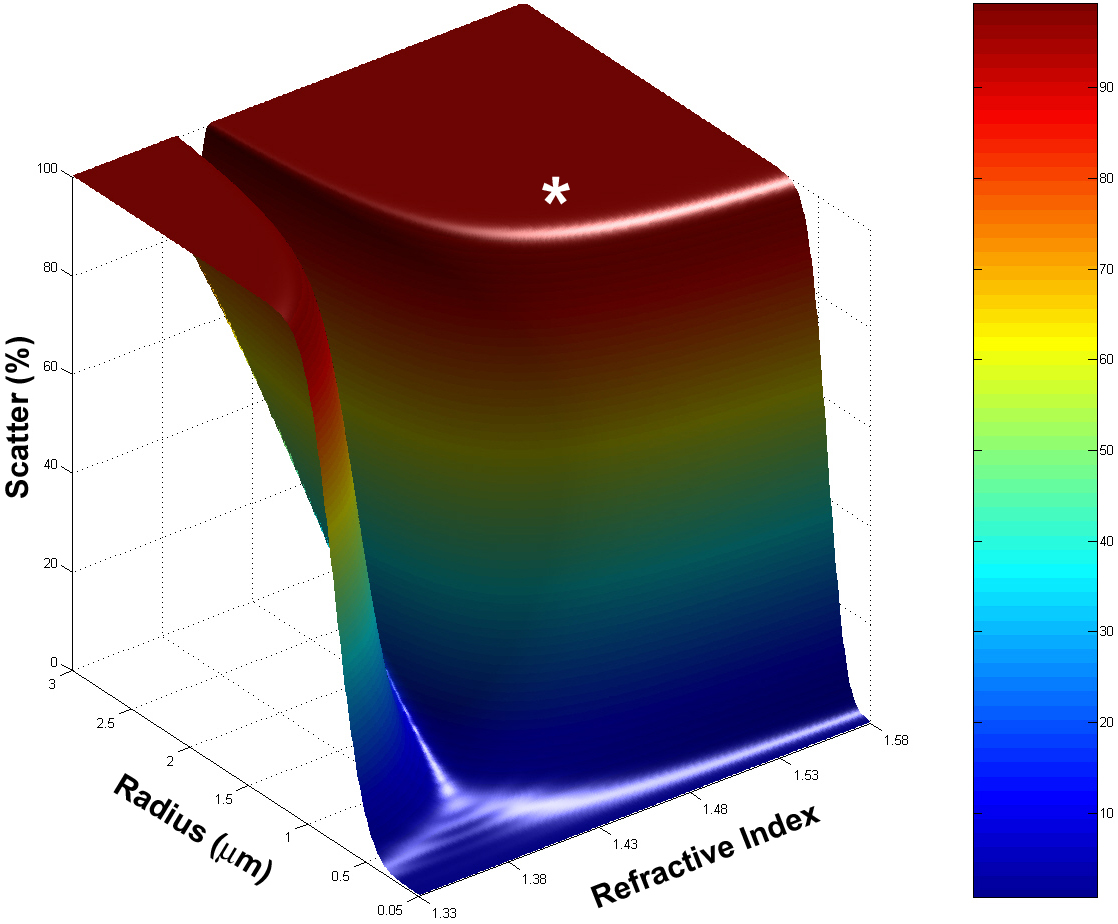
Light scattering by MLBs. In the Indian cataract with the highest density of MLBs, 1,000,000 µm^3^ of tissue was examined by laser scanning confocal microscopy, and in that volume, 412 MLBs were observed. Therefore, the frequency of MLBs was 405,963 per mm^3^ – approximately 100 times that of United States cataracts analyzed in previous studies [32,33]. Mie scattering theory predicts that MLBs contribute significantly to forward scattering despite the fact that they occupy only 0.2% of the volume of the nucleus of the lens. In this case, the lens is almost completely opaque with 99.8% scattering (white asterisk, representing 1.49 refractive index and 2.1 µm average diameter) for 550 nm wavelength light and variable internal refractive index as seen in the surface plot of scattering percent.

In this most exceptional Indian cataract, several individual volumes of tissue were examined by laser scanning confocal microscopy to give a total volume in excess of 1,000,000 µm^3^. In this volume, 412 MLBs were observed. Therefore, the average frequency of MLBs was about 400,000 per mm^3^ – approximately 100 times more than those in United States cataracts analyzed in previous studies [33]. Since MLBs in the exceptional Indian cataract are typically spherical and randomly distributed, the average diameter of 2.1 µm and the frequency of 400,000 per mm^3^ were used to calculate the predicted scattering using Mie theory [34,38]. As in previous studies, the refractive index of the MLB interior and cytoplasm were assumed to be 1.49 and 1.40 [33,34]. Mie scattering theory indicates that MLBs contribute significantly to forward scattering despite the fact that they occupy only 0.2% of the volume of the nucleus of the lens. The MLBs potentially have high scattering efficiencies, and their high density produces a large scattering cross section [34]. With respect to this particular advanced Indian cataract, the lens is almost completely opaque based on predictions for light scattering due to MLBs alone with 99.8% scattering for light of 550 nm wavelength (Figure 7). The light, as it passes through this lens, is predicted to deviate from its original path by scattering angles generally less than 20° for each particle, and multiple scattering events would probably prevent any light from reaching the retina.

## Discussion

The morphological analysis of MLBs in advanced nuclear cataracts and donor lenses from India suggests that the MLBs are similar in size, internal structure, and distribution compared to lenses collected in the United States [32-34]. An important exception was one advanced Indian cataract that displayed a very high density of MLBs. Based on 3D reconstructed volumes from laser scanning confocal serial optical sections, the density of MLBs was about 100 times higher than less mature cataracts from the United States. These data also confirm that MLBs are mainly spherical, demonstrate that the average MLB diameter is 2–3 µm, and show that the MLBs are far apart compared to the wavelength of incident light. The high density of MLBs also provided the opportunity to establish that the MLB distribution was random using rigorous Monte Carlo methods. These properties of the MLBs suggest that they are independent scattering particles and are suitable for light scattering analysis using the Mie theory [34,38-41].

Previous studies have established that Mie’s solution to Maxwell’s electromagnetic equations is an appropriate method for analyzing the contribution to the forward scattering of light by large particles such as MLBs [33,34]. However, these calculations were based on several assumptions and low densities of rare MLBs thus limiting the potential influence of the MLBs to the early stages of nuclear cataract formation and to less than 20% of the incident light. For the high density of MLBs in the exceptional Indian nuclear cataract, the MLBs are calculated to scatter more than 99% of the incident light (Figure 7), therefore, rendering the nucleus totally opaque due to this one structural feature. For these calculations, it was assumed that the internal refractive index of the MLB was 1.49 and the surrounding cytoplasm was 1.40, producing the key input refractive index ratio to the Mie equations as in previous studies [33,34]. This assumed ratio is not as critical for the exceptional Indian nuclear cataract, because if only 20% of the MLBs reached the 1.49/1.40 ratio, then MLBs would still scatter more than 90% of the incident light (data not shown). Recent determinations of nuclear refractive index using magnetic resonance imaging suggest that the value should be near 1.42 [45] instead of 1.40 [46]; however, even if the ratio of 1.49/1.42 were used in the Mie calculations, the scattering would be over 90% (data not shown). Furthermore, other structural features of the nuclear cataract could contribute to the overall opacification including damage to the membranes and to cytoplasmic proteins. For example, the same advanced Indian cataracts prepared for TEM analysis here have been shown to have cytoplasm that can be sufficiently textured to increase the scattering and in some cases produce opacification [47]. Age-related nuclear cataracts most likely have a multi-factorial pathology in which several influences, especially age, nutrition and oxidative stress, may produce multiple structural alterations that contribute to lens opacification. All of the advanced nuclear cataracts from India displayed MLBs, and the high density of MLBs in one cataract suggests that these large particles can play a significant role in mature cataract formation and that efforts to determine how the particles are generated would be valuable.

Although MLB formation is not yet fully understood, many features of this process are evident. Most MLBs appear adjacent to or attached to fiber cell plasma membranes (see Figure 3 and Figure 4), suggesting that the thin multiple layers are derived from a redistribution of membrane lipids and proteins. As discussed previously [32,33], the thinness of the layers precludes the presence of the integral membrane proteins, connexins and aquaporin0, which form gap junctions or water channels, respectively. The absence of integral proteins is also supported by the high intensity of MLB fluorescence using lipid dyes [32,33] and the absence of intramembrane particles in freeze-fracture images of the multilayered coat [48]. Even the spherical nature of the MLBs and the smooth contours of the multilayered coat are consistent with the geometry of pure lipid bilayers, perhaps with a high cholesterol content [49], as opposed to the alternating curvatures in the undulating membrane interdigitations of adult fiber cells [25]. The smooth contours are similar to those of ball-and-socket interdigitations between young cortical fiber cells and may involve the membrane skeleton, except that the simple pinching of a protruding ball would produce a vesicular structure with only one pair of membranes [50]. To achieve the multilamellar pattern, it is likely that more complex processes are involved such as the interleaving of cellular processes from adjacent cells. The formation of complex intercellular processes between human nuclear fiber cells is common, and extensive filopodia-like processes have been reported [51]. An interleaving of cellular processes has been observed in the formation of globular structures resulting from cell breakdown in osmotic cataracts [52]. It is noteworthy for the osmotic stress rabbit model that the cortical fibers, which have flexible membranes and an intact cytoskeleton, form complex interleaving structures whereas the nuclear fiber cells form simpler defects [52]. In both cases, the final result may be similar as the cytoskeleton is broken down, membranes may collapse onto themselves and integral proteins may migrate out of the multilamellar aggregates. As we have suggested previously [32-34], it is likely that the membrane rearrangements leading to MLBs occur when the cells are young, and because the MLBs are exclusively observed in the inner nuclear regions, we hypothesize that the MLBs form in embryonic and fetal development or in the first few years after birth. We further propose that when the MLBs first form, they do not scatter light significantly, but as the lens ages, the MLBs develop into strong scattering sources [32-34]. This hypothesis is consistent with several observations. First, the density of MLBs in most cataracts is low. If the density of MLBs increased with the severity of the nuclear cataract grade, then the advanced Indian cataracts would have had a much higher density but instead were similar to the MLB densities of less mature cataracts from the United States. Second, the exceptional Indian cataract with a high density of MLBs showed a similar cellular structure throughout the inner nuclear region with no obvious cell disruption. Therefore, the random distribution of the high density of MLBs suggests that the number of specific point defects increased rather than a general increase in cell damage or the formation of a region of concentrated damage. Third, the MLBs appear even in transparent donor lenses, suggesting that their formation in the nuclear core involves natural processes of membrane rearrangements during normal development of young fiber cells. Fourth, the suggested early formation of MLBs is consistent with an epidemiological study correlating low weight at one year with the high probability of age-related nuclear cataract formation six decades later [53].

The scattering efficiency of MLBs probably increases as the lens ages. This happens possibly because the lipid coat, which isolates the MLB interior from the cytoplasm, permits the core proteins to condense more than the cytoplasmic proteins thus altering the refractive index ratio. The scattering from the MLBs may therefore not become significant until after the onset of presbyopia and early cataract formation. This suggestion is consistent with the observations that the MLBs appear only in the nuclear core, are too small to be detected by slit lamp examination, contribute mainly to forward scattering, and are very rare in most lenses. Thus, MLBs could exist undetected for decades in nearly all adults. As the MLBs increase their scattering potential with age, the low density in aged transparent lenses limits their impact on image formation, although they could account for the ciliary corona experienced by adults [34,54]. An intermediate density of MLBs, characteristic of most of the nuclear cataracts examined thus far, may cause early visual deficits due to forward scattering and increase the probability of age-related nuclear cataract formation. A high density of MLBs as reported here for the exceptional Indian cataract may foreshadow early serious visual deficits, early nuclear cataract formation, and total lens opacification.

There are several possible explanations for the high density of MLBs in the exceptional Indian cataract. The high MLB density may result from an increase in oxidative stress during fetal development or at birth. It should be noted that in the rat animal model for oxidative stress, increased lipid peroxidation was detected throughout the lens, and the lens nuclear core was the only location for the formation of multilamellar cellular debris similar to the MLBs [55]. Another possibility is that there may have been a metabolic imbalance where an excess of lipid over protein may result in excess membrane during rapid lens growth in early development. The response of the lens may be to sequester excess membrane lipid into focal defects that eventually transform into the MLBs. A third possibility is that there may have been a minor and as yet unidentified genetic defect that influences early fiber cell elongation. Although it is not possible at present with one example to determine the underlying pathology, it is clear that if the MLBs are formed early in development, then the high density of MLBs may provide an explanation for the early onset and rapid development of blinding cataract in the Indian population. In future studies, light scattering data on intact lenses must be collected before the tissue is analyzed for pathology so that in vivo clinical conditions may be correlated with in vitro analysis of cells and MLB density. With these advances, the contribution of MLBs to forward scattering and cataract formation can be confirmed.
